# Multiple tumorous lesions of the pituitary gland

**DOI:** 10.1007/s42000-022-00392-9

**Published:** 2022-08-10

**Authors:** Jannik von Schöning, Jörg Flitsch, Dieter K. Lüdecke, Rudolf Fahlbusch, Michael Buchfelder, Rolf Buslei, Ulrich J. Knappe, Markus Bergmann, Walter J. Schulz-Schaeffer, Jochen Herms, Markus Glatzel, Wolfgang Saeger

**Affiliations:** 1 Institute of Neuropathology, University of Hamburg, UKE, Martinistraße 52, 20246 Hamburg, Germany; 2 Clinic of Neurosurgery, University of Hamburg, UKE, 20246 Hamburg, Germany; 3 International Neuroscience Institute (INI), Rudolf-Pichelmayr-Str. 4, 30625 Hannover, Germany; 4 Clinic of Neurosurgery, Friedrich-Alexander University Erlangen-Nürnberg (FAU), 91054 Erlangen, Germany; 5 Institute of Pathology, SozialStiftung Bamberg, 96049 Bamberg, Germany; 6 Department of Neurosurgery, Johannes-Wesling-Klinikum Minden, 32429 Minden, Germany; 7 Institute of Neuropathology, Klinikum Bremen-Mitte, 28205 Bremen, Germany; 8 Institute of Neuropathology, University of Saarland, 66421 Homburg, Germany; 9 Zentrum für Neuropathologie und Prionforschung, LMU-University of Munich, 81377 Munich, Germany

**Keywords:** Pituitary, Adenoma, Multiple tumorous lesions, Double adenomas, PANCH, Gangliocytoma, Metastasis, Pituitary cysts, Rathke’s cleft cyst, Spindle cell oncocytoma, Pituicytoma, Granular cell tumor, PitNETs

## Abstract

**Purpose/Objective:**

Multiple tumorous lesions in one pituitary gland are rare and mostly described in case reports. Their incidences and combinations are defined in larger collectives. Therefore, we analyzed our large collection for double tumors and combinations of tumors, cysts, and inflammation.

**Methods:**

The German Registry of Pituitary Tumors, including cases from 1990 to 2018, served as the database. Our collection comprises a total of 16,283 cases up until the end of 2018. Of these cases, 12,673 originated from surgical and 3,610 from autopsy material. All specimens were fixed in formalin and embedded in paraffin. The sections were stained with hematoxylin–eosin and PAS. Monoclonal (prolactin, TSH, FSH, LH, and α subunit) or polyclonal (GH and ACTH) antibodies were used to detect pituitary hormones in the lesions. Since 2017, antibodies against the transcription factors Pit-1, T-Pit, and SF-1 have been used in difficult cases. The criteria of the 2017 WHO classification have been basic principles for classification since 2018 (Osamura et al. 2017). For differentiation of other sellar tumors, such as meningiomas, chordomas, or metastases, the use of additional antibodies was necessary. For these cases, it was possible to use a broad antibody spectrum. Autopsy pituitaries were generally studied by H&E and PAS sections. If any lesions were demonstrated in these specimens, additional immunostaining was performed.

**Results:**

Multiple tumorous lesions with more than one pituitary neuroendocrine tumor (PitNET) respectively adenoma make up 1.4% (232 cases) in our collection. Within the selected cases, synchronous multiple pituitary neuroendocrine tumors (PitNETs) account for 17.3%, PANCH cases (pituitary adenoma with neuronal choristoma) for 14.7%, PitNETs and posterior lobe tumors for 2.2%, PitNETs and metastases for 5.2%, PitNETs and mesenchymal tumors for 2.6%, PitNETs and cysts for 52.2%, and PitNETs and primary inflammation for 6.0%. The mean patient age was 53.8 years, with a standard deviation of 18.5 years. A total of 55.3% of the patients were female and 44.7% were male. From 1990 to 2018, there was a continuous increase in the number of multiple tumorous lesions.

**Conclusion:**

From our studies, we conclude that considering possible tumorous double lesions during surgeries and in preoperative X-ray analyses is recommended.

## Introduction

In the past, in the vast majority of surgical specimens of the pituitary, the histopathological examination revealed only one lesion [1]. Since in a small number of cases, however, a second lesion was found to coexist, in 2017, the diagnosis of synchronous multiple adenomas—now known as PitNETs according to the International Pituitary Pathology Club—was included in the WHO classification [2]. In the present study, we preferentially use the term PitNET instead of adenoma, as do many other authors [3, 4].

Multiple lesions refer to two or more morphologically separated lesions. In the pituitary gland, pituitary neuroendocrine tumors are the most common neoplasms [5]. The secondary lesion coexisting with a PitNET can be neoplastic, vascular, congenital, or inflammatory [6].

So far, there is no agreed upon pathogenic explanation for the coexistence of two separate lesions. Since many cases of double lesions have been described in the literature, random occurrence is unlikely.

The identification of two distinctly different tumorous lesions in the pituitary is clinically important, since the different lesions may cause different symptoms. For example, there were two patients with Cushing’s disease in our collection whose surgical specimens harbored a sparsely granulated prolactin PitNET. The simultaneously present ACTH-secreting PitNET was not identified until the second operation. Clinical signs of two different PitNETs or tumors during surgery may be differences in location, consistency, and demarcation. Radiological signs are inconclusive [29].

The aim of our study was to describe the different tumorous lesions coexisting in the pituitary, especially their types and numbers, with the goal of determining whether there were typical tumor combinations or if the combinations were only random. Possible pathogenetic mechanisms will be discussed.

## Materials and methods

From the German Registry of Pituitary Tumors, we extracted the cases from the years 1990 to 2018. This yielded a total of 16,283 cases. The registry, founded by one of the authors (WS) in 1970, collects all surgical and autoptical pituitary specimens sent to the Institute of Pathology of the University Hospital Eppendorf, Hamburg, Germany (1970 to 1978 and since 2013), or to the Institute of Pathology of the Marienkrankenhaus, Hamburg, Germany (1979 to 2012) for histopathological assessment. In 1995, it was officially incorporated into the German Society of Endocrinology as the German Registry of Pituitary Tumors. With a total of 16,283 cases by the end of 2018, it represents an incomparably large collective. Of these cases, 12,673 originate from surgical and 3610 from autopsy material. Since the existing literature mainly describes individual cases, the size and time frame of our collective are of particular interest.

During histopathological preparation, the specimens were fixed in formalin and embedded in paraffin. The sections were stained with hematoxylin–eosin and PAS.

Monoclonal antibodies for prolactin, TSH, FSH, LH, and α subunit and polyclonal antibodies for GH and ACTH were used to detect pituitary hormones in the lesions. We used the PAP method. Double immunostaining was not performed. Ki-67 was determined in all tumors and in many of them also p53.

PitNETs were classified according to the WHO classification current at that time, and mainly to that of 2004 [7]. The criteria of the 2017 WHO classification have been the basic principles for classification since 2018 [2]. Since that time, antibodies against the transcription factors Pit-1, T-Pit, and SF-1 have been used in difficult cases, especially if the hormone expression or histologic structures of the PitNET do not correlate with clinical hyperfunction, or if extremely unusual structures or combinations of hormone expression exist.

In many cases, the use of additional antibodies was necessary to identify other sellar tumors, such as meningiomas, chordomas, or metastases. A wide spectrum of antibodies would then be applied.

Pituitaries obtained during autopsies were generally studied in H&E- and PAS-stained sections. Additional immunostaining methods were performed if any lesions were found in these sections.

## Results and discussion

From 1990 to 2018, a total of 232 cases (1.4% of the collection) with multiple tumorous lesions were selected from the registry. Of these, 38 were double PitNETs, two triple PitNETs, 34 PitNETs associated with a gangliocytoma, five PitNETs with neurohypophyseal tumors, and 12 PitNETs neighboring or within metastases; six PitNETs were accompanied by mesenchymal tumors and 121 PitNETs by cysts, while 14 PitNETs were seen in connection with primary inflammations.

In total, 79% of all cases in the registry were associated with PitNETs.

Of these cases, 2.04% were combined with another lesion. In the past few decades, there has been a slight increase in the number of diagnoses of multiple lesions. In percentages, more double lesions were diagnosed in post-mortem material (1.63%) than in the surgical material (1.36%) (Table 1).

**Table 1:** Multiple lesions in surgical and autopsy material

Cases (surgical and autopsy material)	Number	Autopsy ([%] of all autopsy cases)	Surgical ([%] of all surgical cases)
Double PitNETs	38	21 (0.58)	17 (0.13)
Triple PitNETs	2	2 (0.06)	0 (0)
PitNET and gangliocytoma (PANCH)	34	0 (0)	34 (0.27)
PitNET and posterior lobe tumor	5	2 (0.06)	3 (0.02)
PitNET and metastasis	12	6 (0.17)	6 (0.05)
PitNET and meningioma	6	2 (0.06)	4 (0.03)
PitNET and cysts	121	25 (0.69)	96 (0.76)
PitNET and primary inflammation	14	1 (0.03)	13 (0.1)
Total	232	59 (1.63)	173 (1.36)

The average age of patients at the time of surgery was 53.8 years, with a standard deviation of 18.5 years. A total of 45% of the patients were male and 55% female.

Double PitNETs were found quantitatively similarly frequently in post-mortem and surgical material. However, there was a higher proportion of cases in the autopsy specimens than in the surgical ones (0.58% vs. 0.13%) (Table 1). All cases of PitNET together with a gangliocytoma were diagnosed in the surgical material.

The most common tumor type in double lesions is the PitNET of sparsely granulated prolactin cell type (22.5%). As opposed to the tumor registry (8.9%), prolactin-secreting PitNETs in multiple lesions are more common. Null cell adenomas accounted for 21.1% in our study, although this proportion would be much lower according to the current WHO classification due to its new definition of null cell adenoma [2].

We also found that gonadotrophic PitNETs represented the largest proportion in the tumor registry at 31.0%, although they were present in only 16.7% of the multiple lesions.

ACTH-secreting PitNETs represented 12% of the adenomas in multiple lesions, while their percentage in the tumor registry is 16.2%. GH-secreting PitNETs and mixed GH-/prolactin-secreting PitNETs were found in 23.3% of the PitNETs in double lesions and make up 21.47% of the entire tumor registry.

### Multiple PitNETs

Thirty-eight cases (21 post-mortem and 17 surgical specimens) with multiple PitNETs were described in the German Registry of Pituitary Tumors. Significantly more men (68%) were affected by double PitNETs, and 0.5% of all PitNETs in the database coincided with a second PitNET.

In the group of double PitNETs, sparsely granulated prolactin cell tumors made up the largest proportion (25.0%).

The cross table (Table 2) shows the combination of types of PitNETs in the 76 pituitaries with multiple PitNETs. In 10 of these glands, both PitNETs were from the same cell line. In 36 PitNETs, the cell lines were clearly different, and in another 30 PitNETs, it was not possible to determine the cell line with certainty. The cases that could not be determined with certainty due to lack of determination of transcription factor contained null cell adenomas.

**Table 2:** Combinations of transcription factors in multiple PitNETs

Cell line of first PitNET [*N*]	Cell line of second PitNET [*N*]				Total
	T-PIT	PIT-1	SF-1	Not definable	
T-PIT	0	7	3	2	12
PIT1	7	10	8	8	33
SF1	3	8	0	1	12
Not definable	2	8	1	8	19
Total	12	33	12	19	76

The results of Pearson’s chi-square test support the null hypothesis that there is no significant correlation between the cell line combinations (chi-square test, *p* = 0.063, *n* = 76).

Clinical data were available in 33 of 38 cases. In 14 cases, the PitNETs were clinically inactive, in nine cases acromegaly was present, in two cases increased IGF-1 levels were documented, in six cases hyperprolactinemia was present, and in two cases the patients suffered from Cushing’s disease.

Women diagnosed with a double PitNET showed clinical signs of acromegaly as well as elevated IGF-1 levels in 41.3% of the cases. Cushing’s disease was present in 16.7% of the women.

Men with a double PitNETs had acromegaly with elevated IGF-1 levels in 24.0% of the cases. In 36% of the cases, the PitNETs were inactive, while hyperprolactinemia was detected in 20%.

Three instances of a pituitary with three separate, simultaneous PitNETs cases were identified at autopsy. In one, there were three individual null cell adenomas, in the second two null cell adenomas and a TSH tumor coexisted, and in the third there were two LH cell tumors and a null cell adenoma. All nine PitNETs were microadenomas (maximum diameter less than 1 cm) which were completely separated from each other.

Of the total of 85 multiple PitNETs, 11.8% are combinations of tumors with the same transcription factor, 42.4% are tumor combinations with different transcription factors, and 35.3% have a null cell adenoma in the tumor combination: it was therefore not possible to make a statement regarding the cell line since the transcription factor could not be determined (Table 3).

**Table 3:** Combinations of multiple PitNETs

	PitNETs [*N*]	Proportion [%]	Cases [*N*]
Triple PitNETs of the same subtype	3	3.5	1
Triple PitNETs (2 × same subtype, 1 × different subtype)	6	7.0	2
Double PitNETs with the same transcription factor	10	11.8	5
Double PitNETs with different transcription factors	36	42.4	18
Double PitNETs with unidentifiable transcription factors	30	35.3	15
Total	85	100	41

Atypical PitNETs (classification before 2017), respectively aggressive PitNETs identified by adenoma type, increased Ki-67 index, rate of mitosis, and invasion (classification after 2017) were not significantly more frequent in PitNET combinations. The Knosp grades for differentiation of macroadenomas from microadenomas were unknown to us in most cases.

Studies on surgical material report prevalence at between 0.3 and 1.3% for multiple PitNETs [8–10]. In our collective, multiple PitNETs account for 0.66% of all PitNETs. Therefore, our data are within the range reported in the literature. In our unselected autopsy series, multiple PitNETs occur with a prevalence of 0.58%. Comparable studies report a slightly higher prevalence of 0.9% [11].

In our study, men were affected significantly more often than women (exact binominal test, two-sided: *p* = 0.047). However, similar studies have found a balanced sex ratio [12] or even female predominance [13]. Based on these data, the male predominance in our study could be a random occurrence.

Reports describe GH-secreting PitNETs as being most frequently diagnosed in pituitaries with multiple PitNETs [10, 11]. Recent studies described increasing numbers of ACTH-secreting PitNETs in patients with multiple PitNETs [15]. Because of the large number of GH- and ACTH-secreting tumors, multiple PitNETs are often associated with the symptoms of acromegaly or Cushing’s disease [12]. In the surgically removed specimens in our collection, symptoms of acromegaly were described in 64.7% and symptoms of Cushing’s disease in 11.8% of the cases. Due to the high prevalence of acromegaly in multiple PitNETs, it may be useful to perform IGF-1 screening when multiple PitNETs are suspected.

Multiple PitNETs have been listed in the WHO classification of endocrine tumors since 2017 [2], but the tumors are required to consist of different cell lineages to be classified as such.

If it can be determined from the specimens that the two coincident lesions coexist separately from each other, the cases in which the same cell lineage is present should also be listed as multiple PitNETs. However, it is doubtful whether these lesions may actually be considered true multiple PitNETs.

Etiologically, there are different theories for the genesis of multiple PitNETs. In the multi-hit theory, for example, it is hypothesized that two mutated monoclonal pituitary cell types coincidentally give rise to two tumors that develop simultaneously [14]. Other authors propose that one PitNET can induce the development of another via growth factors [6]. This theory is especially associated with GH-secreting tumors [9].

In summary, the etiology of multiple PitNETs is thought to resemble that of solitary PitNETs. Therefore, it is still most likely that multiple PitNETs are formed by random events. This hypothesis is also supported by the data we selected.

### Adenoma and gangliocytoma—PANCH

Thirty-four instances of the combination of a PitNET and a gangliocytoma, all identified in surgical specimens, were found in the database. There were significantly more women (76.5%) affected by pituitary adenoma with neuronal choristoma (PANCH) than men (20.6%) (exact binomial test, two-sided, *p* = 0.001, *n* = 33). The majority of the PANCH cases (88.2%) were associated with a GHRH-containing gangliocytoma. Of the 34 identified cases, 31 tumors were PIT-1 positive. Two PitNETs originated from the T-Pit cell line, and in one case, it was not possible to determine the cell line because it was a null cell adenoma (Table 4). The proportion of Pit-1-positive tumors was 91.2%.

**Table 4:** Combinations of PitNET and gangliocytoma type

PitNET subtypes (surgical material)	Gangliocytoma	Number	Percent [%]
Sparsely granulated GH	GHRH	9	26.5
Mixed GH/prolactin	GHRH	20	58.8
Sparsely granulated prolactin	Not reliably determinable	2	5.9
Densely granulated ACTH	CRH	1	2.9
Densely granulated ACTH	Not reliably determinable	1	2.9
Null cell tumor	GHRH	1	2.9
Total		34	100

Acromegaly was clinically diagnosed in 82.4% of the PANCH cases. Cushing’s disease was present in 2.9% and hyperprolactinemia in 5.9%, and the clinical presentation was unremarkable in 8.8%.

PANCH cases are of special interest because of the smooth transition between the lesions and the influence of the origin of the lesions on each other. Current studies suggest that transdifferentiation from a PitNET is responsible for the development of a PANCH [15]. If this is confirmed, it will not be possible to classify these lesions as multiple lesions.

The prevalence of PANCH cases in the tumor registry was 0.21%. Yang et al. reported a PANCH prevalence of 0.46% in their collective of 4317 cases [16]. Other studies reported prevalence values ranging from 0.52 to 1.26% [17, 18]. These studies examined only surgically removed specimens. Since all instances of PANCH in our study were found in surgical material, the prevalence may also only be related to the surgical database, resulting in a slightly increased prevalence of 0.26%.

We found significantly more women (78.8%) with PANCH than men (exact binomial test, two-sided, *p* = 0.001), this being similar to the findings of Yang et al. (65% female) [16]. The reason for this distribution is still unknown. No skewed sex ratios were found in similar studies of acromegaly [19].

Although we diagnosed acromegaly in 82.4% of our PANCH cases, the proportion of acromegaly cases is lower in comparable studies. According to the study of Yang et al., 75% of PitNETs were sensitive to GH, while only 25% were clinically diagnosed with acromegaly [16]. Most of these PitNETs are of the mixed, sparsely granulated GH/prolactin type [15, 20, 21].

**Fig. 1:**
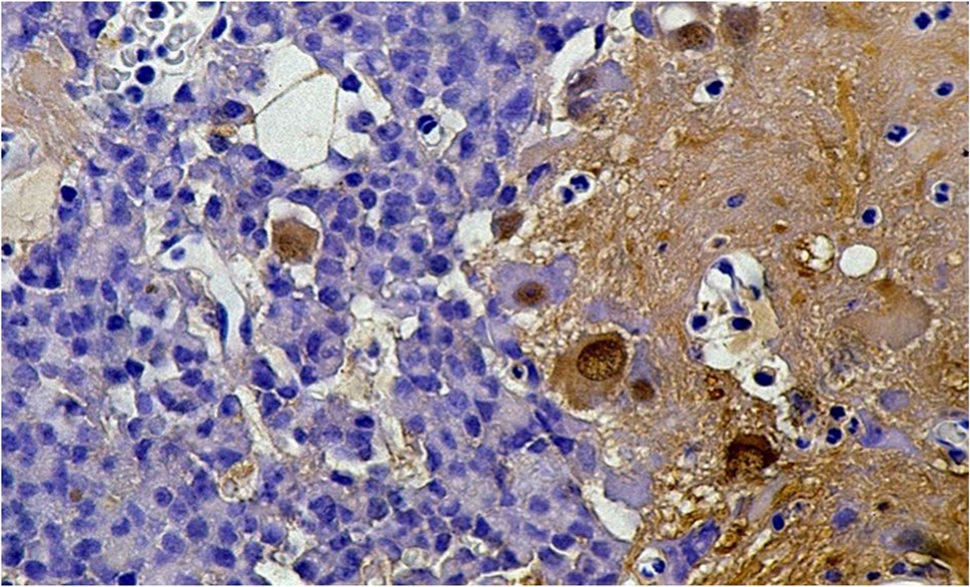
Sparsely granulated GH PitNET and GHRH gangliocytoma. GHRH gangliocytoma (right) with loosely positioned ganglionic cells (deep brown) together with an GH-secreting PitNET (left), blurred from the gangliocytoma; GHRH; 440: 1

It is likely that the two lesions affect each other pathogenetically due to the releasing factor of the gangliocytoma [17, 20, 22]. The close relationship between the two lesions is also indicated by the possible transition of gangliocytoma cells into PitNET cells and vice versa (Fig. 1) [15, 20]. According to reports in the more recent literature [23, 24], it may be more likely that mixed gangliocytoma and PitNET may originate by transdifferentiation of a previous PitNET, since monoclonality of adenoma and neuron-like cells was demonstrated by the X-chromosome inactivation method [23].

Only 5.6% of gangliocytomas in the tumor registry are solitary. Published reports estimate that 65–85% of all pituitary gangliocytomas coexist with a PitNET [25].

### PitNET and posterior lobe tumor

Five cases with a combination of a posterior lobe tumor and a PitNET were found in the database.

Of the posterior lobe tumors, 8.3% of spindle cell oncocytomas, 10% of pituicytomas, and 11.1% of granular cell tumors were associated with a coexistent adenoma. In total, 10% of all posterior lobe tumors in the tumor registry coexisted with an adenoma (Table 5).

**Table 5:** Posterior lobe tumors in the German Registry of Pituitary Tumors

Posterior lobe tumor	Cases from 1990 to 2018	% of non-PitNETs (*n* = 1,953)	% of all cases (*n* = 16,283)	% in combination with a PitNET (cases)
Spindle cell oncocytoma	12	0.61	0.07	8.33 [1]
Pituicytoma	20	1.02	0.12	10.0 [2]
Granular cell tumor	8	0.92	0.05	11.1 [2]
Total [*N*]	40	2.56	0.24	12.5 (5)

We found the following combinations:

- PitNET sparsely granulated GH and a granular cell tumor
- PitNET sparsely granulated prolactin and a granular cell tumor
- PitNET sparsely granulated prolactin and a pituicytoma
- Null cell adenoma and a pituicytoma
- Null cell adenoma and a spindle cell oncocytoma

PitNETs associated with posterior lobe tumors are very rare, though they are probably underdiagnosed processes in our tumor registry as well as in the literature. The combination of a neurohypophyseal tumor and a PitNET was first described by Neidert et al. in 2016 [26]. In the German Registry of Pituitary Tumors, the first instance of this combination was documented in 2007.

Due to the posterior position of the neurohypophyseal tumor, it may not be detected during surgery. Furthermore, in most of the cases, a second lesion besides the PitNET was not detected in the preoperative MRI [27]. A total of 10% of all posterior lobe tumors in the tumor registry are associated with a coexistent PitNET. In most cases, the PitNET has a greater biological impact than the posterior lobe tumor because of its hormone expression (27).

### PitNET and metastasis

Twelve cases of combination of a PitNET and a metastasis were identified. Six cases were diagnosed in surgical and six in post-mortem specimens. The patients (nine male and three female) with this tumor combination had an average age of 70.3 years.

There were 112 cases of pituitary metastasis in the database, which yields a 10.7% incidence of a coexisting PitNET.

Five metastases originated from a lung tumor, two from a kidney tumor, and one each from a malignant melanoma, a prostate carcinoma, and a tumor of the nasal sinus. In one instance, the origin was unknown (Table 6).

**Table 6:** Combination of PitNET and metastasis

PitNET subtypes	Examination material	Localization	Metastasis	Origin
Densely granulated GH tumor	Surgical	PitNET	Adenocarcinoma	Sinunasal
GH/prolactin tumor	Post-mortem	Posterior lobe	Malignant melanoma	Skin
Sparsely granulated prolactin tumor	Surgical	PitNET	Undifferentiated carcinoma	Unknown
Sparsely granulated prolactin tumor	Post-mortem	Capsule	Small cell carcinoma	Lung
Densely granulated ACTH tumor	Surgical	PitNET	Clear cell carcinoma	Kidney
Densely granulated ACTH tumor	Post-mortem	Capsule	Scirrhous carcinoma	Gastric
FSH/LH-, FSH-, or LH-tumor	Surgical	PitNET	Adenocarcinoma	Prostate
FSH/LH-, FSH-, or LH-tumor	Post-mortem	Posterior lobe and sinus cavernosus	Squamous cell carcinoma	Lung
FSH/LH-, FSH-, or LH-tumor	Surgical	PitNET	Clear cell carcinoma	Kidney
FSH/LH-, FSH-, or LH-tumor	Surgical	PitNET	Adenocarcinoma	Lung
Null cell tumor	Post-mortem	Capsule	Undifferentiated carcinoma	Lung
Plurihormonal tumor	Post-mortem	Capsule, anterior lobe and in PitNET	Small cell carcinoma	Lung

**Fig. 2:**
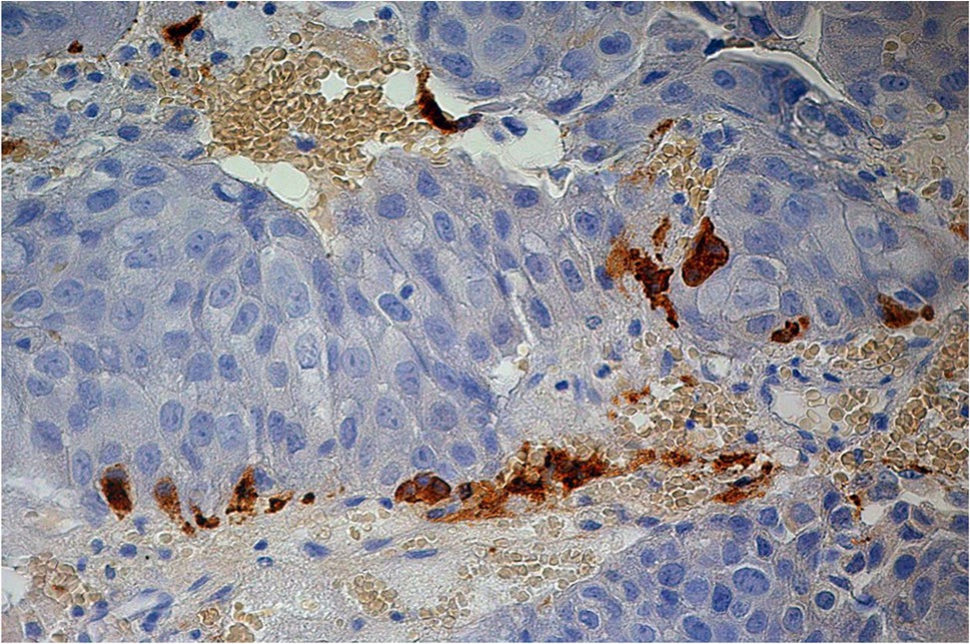
Poorly differentiated bronchopulmonary adenocarcinoma with gonadotropic adenoma. Large metastatic tumor within a densely granulated GH PitNET present only in very small remnants of PitNET cells (deep brown); GH; 500:1

In these twelve cases, seven of the metastases were found within the coexisting PitNET. In the histopathological sections, the border between the PitNET and the metastasis was indistinct (Fig. 2). It is remarkable that in all surgical cases the metastases were found within the PitNET.

In a further three cases, the metastasis was in the capsule and, in two cases, in the posterior lobe.

Accordingly, in 58% of cases, the metastasis was localized within the PitNET and in 42% it was separated from the PitNET (Table 6), a percentage difference which is not statistically significant (exact binomial test, bilateral, *p* = 0.774, *n* = 12).

Metastases account for up to 3.6% of post-mortem specimens, while metastases that are removed surgically account for about 1% of all pituitary tumors [2].

In total, 10.7% of the pituitary metastases in the German Registry of Pituitary Tumors coexist with a PitNET. The first two cases of this coexistence were reported in 1985 [28].

In 2009, Hoellig et al. reviewed 15 cases of a metastasis inside a PitNET from their own material and from published accounts. The study included only cases with metastases in the PitNET. They hypothesized that the metastasis followed the blood supply of the PitNET [29].

A further theory of pathogenesis proposed that the primary tumor initially metastasizes to the surrounding bone and, presumably, spreads through local neovascularization to the pituitary gland. With a pre-existing PitNET, the primary tumor is more likely to metastasize into the PitNET. In 58% of our cases, the primary tumor metastasized to within the PitNET. In the surgical specimens, this rate was 100%. The reason for this is most probably the changed formations of capillaries inside the PitNET. In addition, it is hypothesized that the tissue around the PitNET has a certain resistance due to the pre-existing lesion [30]. This theory is supported by the fact that breast, prostate, kidney, and lung cancers often metastasize to the surrounding bones and from there to the pituitary gland [31–33]. Metastases of these primary tumors were also identified in our study.

In our own separate collection of 153 post-mortem pituitaries and their surrounding bone structures, metastases were found in the sellar bone in 29 cases (18.9%) [34]; 76% of these corresponded to metastases from carcinomas and 24% were infiltrating hematological malignancies. The origins of the sellar metastases were prostate cancer (17.2%), breast cancer (17.2%), lung cancer (20.7%), stomach cancer (7%), urinary bladder cancer (7%), ovarian cancer (3.4%), and colorectal cancers (3.4%). The involved hematological malignancies were plasmacytoma (1.3%), malignant non-Hodgkin’s lymphomas (2.0%), and Hodgkin’s lymphoma (8.5%). Most metastases were identified in the pituitary capsule, followed by the posterior and the anterior lobe. All instances of pituitary metastasis were combined with and derived from bone metastases.

### PitNET and cysts

In unselected autopsy material, Rathke’s cleft cysts occur in up to 33% of pituitaries and comprise the most common secondary lesion found together with the PitNET [35]. Nevertheless, the coexistence of Rathke’s cleft cysts and PitNETs is rarely described despite this high incidence [6].

In our database, 16.6% of all Rathke’s cleft cysts in the tumor registry were associated with a PitNET (Table 7). At the same time, only 0.64% of all PitNETs were associated with a Rathke cleft cyst. Noh et al. reported a similar incidence of 0.51% in their study [36]. Sumida et al. even found that 3.5% of all PitNETs were associated with Rathke’s cleft cyst, but a comparison with the results of our study is difficult because they only evaluated radiological data [37].

**Table 7:** Cysts in the German Registry of Pituitary Tumors from 1990 to 2018

Type of cyst	Number	% of non-PitNETs (n=1,953)	% of all cases (n=16,283)	Cases in multiple lesions (proportion of subgroup in relation to the total collective [%])
Rathke’s cleft cyst	441	9.03	2.71	73 (16.50)
Colloid cyst	66	1.35	0.41	5 (7.58)
Arachnoid cyst	21	0.43	0.13	2 (9.52)
Unclassifiable cyst	30	0.61	0.18	11 (36.67)
Total	583	11.93	3.59	91 (15.61)

As the embryological origin of both the anterior pituitary lobe and Rathke’s cleft cyst is Rathke pouch, a combined origin may be hypothesized [38]. In further research, cases with a Rathke cleft cyst within the PitNET may be of interest to determine whether the two lesions have a common origin (proportion in our collective: 5.5%).

In addition, Ikeda et al. proposed a mechanism in which an existing Rathke cleft cyst could be a risk factor for a PitNET. Furthermore, a ruptured Rathke cleft cyst is more likely to be associated with a PitNET than an unruptured Rathke cleft cyst [39].

To the best of our knowledge, the combination of a colloid cyst and a PitNET has not yet been described. In our study, 7.6% of the colloid cysts in the tumor registry were adjacent to a PitNET (Table 7). The pathogenesis of colloid cysts is not fully clarified. It is thought that colloid cysts arise by natural cell death, and that small cysts then arise around this area [40]. The coexistence with a PitNET is believed to be a fortuitous event.

Arachnoid cysts are very rare lesions, and we found only 21 cases in our tumor registry (0.13%). A coexistent PitNET was present in two of these cases (9.5%) (Table 7). Pathogenetically, it is postulated that the mesencephalic membrane often has a perforation [41], whereby a second lesion (such as a PitNET) may form a valve on this membrane. By dividing the membrane, a cyst can arise from the roof of the diencephalon membrane and the base of the mesencephalon membrane [42]. This theory could explain the interacting coexistence of an arachnoid cyst and a PitNET.

### PitNET and meningioma

Six cases of meningioma and PitNET were identified in the studied material. In the pituitary tumor registry, meningiomas account for 0.9% of all cases. Four percent of all diagnosed meningiomas—one of which was malignant—were accompanied by a coexistent PitNET.

Three meningiomas found together with a PitNET were WHO grade I meningiomas.

No abnormal frequency was found in the PitNET distribution.

Four percent of the sellar meningiomas in the tumor registry coexisted with a PitNET. Similar studies of coexisting lesions of PitNETs and meningiomas found that GH-secreting PitNETs were most frequently present together with meningiomas (30%) [43, 44].

It might therefore be likely that growth factors from the PitNET enhance meningioma development [44, 45]. Friend et al. reported in 1999 that GH and IGF-1 significantly increase the growth rate of meningiomas [45]. In our study, there was no PitNET preference.

Moreover, we considered only those cases in which the meningioma had a sellar location. However, the study by Zhu et al. also showed that meningiomas, as primary brain tumors, do not require a close positional relationship to the PitNET for there to be a suspicion of a coherent pathogenetic relationship, as the incidence of meningiomas in the normal population (6/100,000) was significantly lower in the subjects of their study than in patients with a pre-existing PitNET (7/1000). Zhu et al. indicated that germline mutations in MEN1 may be associated with the tumorigenesis of PitNETs with meningiomas [46]. Other studies reported the characterization of a CSC-like subpopulation in meningiomas and PitNETs, suggesting that these tumors may be related to the activity of stem cell-like subpopulations [24].

### PitNET and primary inflammation as tumor-like lesions

In our series from 1990 to 2018, we identified 14 instances with the combination of primary inflammation and PitNET. The sources of the inflammation in these 14 cases were lymphocytic hypophysitis (*n* = 9), granulomatous hypophysitis (*n* = 3), and one case each of abscess and xanthogranuloma.

Six cases were clinically inactive, compression symptoms were present in three cases, and in one case, the PitNET was hormonally active (TSH-secreting PitNET). The two GH-secreting PitNETs were associated with acromegaly.

Primary inflammation of the pituitary in addition to PitNETs is rarely described in the literature. Metastudies by Sivakoti et al. [47] identified nine cases of primary inflammation combined with a PitNET. In four cases, the inflammation was granulomatous and in five cases lymphocytic. Fourteen PitNETs with primary inflammation were listed in the tumor registry [48], of which 64.3% were lymphocytic and 21.4% granulomatous inflammation types.

In 1983, Holck et al. were the first to propose various theories for the coexistence of a PitNET and inflammation [49]. They hypothesized random coincidence though also theorizing that the PitNET could trigger the inflammation by producing specific substances. Similar hypotheses have been published by other authors [47, 50]. Yamamoto et al. stated that the transcription factor PIT-1 is associated with hypophysitis [51]. Since 50% of our cases had PIT-1-positive PitNETs, a link may be possible. In the study by Sivakoti et al., one-third of coexisting PitNETs were PIT-1-positive [47].

## Conclusion

Identifying the existence of double or multiple pituitary lesions is becoming increasingly important in histopathology and, as a result, also in the preoperative diagnosis. If the clinical symptoms do not change after surgery, or if the histopathological specimens do not match the clinical findings, another undetected lesion might be the reason. Identification of multiple lesions can also provide information on the pathogenesis of solitary lesions. Their detection therefore represents a vital research contribution to tumorigenesis.

The reported prevalence of PitNETs in the histopathological specimens of unselected post-mortem pituitaries is between 10 and 14.4% [52, 53].

If this prevalence is applied to the 3610 autopsy cases in the pituitary tumor registry, 361 to 520 PitNET cases would be expected in the group of post-mortem specimens. Since in our study 59 autopsy cases were associated with multiple lesions, this would correspond to a prevalence of 11.3 to 16.3% for the presence of a coexistent secondary lesion in addition to a PitNET, using the data from studies by Ezzat et al. and Buurman and Saeger [52, 53]. Consequently, because of these large percentages, the actual number of multiple lesions might be higher than that reported in our data and in the literature.

The latter observation underlines the importance of localization of double or multiple lesions in the clinical and histopathological routine, as one tumor can be active and the coexisting one inactive. Multiple lesions can be suspected if there is a difference in consistency and demarcation in the X-ray. Whether these two or more lesions occur together randomly or if they arise from a single origin needs to be examined more closely.

Although individual cases of a craniopharyngioma coinciding with a PitNET have been described, we were unable to detect this combination in our collective [54–57], thus showing the rarity of this coexistence.

Based on our studies, we can conclude that it is important to take the possibility of tumorous double or multiple lesions into consideration when interpreting preoperative radiographs and also during the operation itself. This is especially essential in patients who exhibit more complex anatomical locations of the lesions.
